# Induction Fluorouracil-Based Chemotherapy and PET-Adapted Consolidation Chemoradiation with Esophagectomy for High-Risk Gastroesophageal Adenocarcinoma

**DOI:** 10.3390/cancers15174375

**Published:** 2023-09-01

**Authors:** Andrew J. Sinnamon, Rutika Mehta, Samir Saeed, Gregory Y. Lauwers, Russell F. Palm, Jessica M. Frakes, Sarah E. Hoffe, Jobelle J. Baldonado, Jacques P. Fontaine, Jose M. Pimiento

**Affiliations:** 1 Department of Gastrointestinal Oncology, H. Lee Moffitt Cancer Center, 12902 Magnolia Drive, Tampa, FL 33612, USA; rutika.mehta@moffitt.org (R.M.); samir.saeed@moffitt.org (S.S.); gregory.lauwers@moffitt.org (G.Y.L.); russell.palm@moffitt.org (R.F.P.); jessica.frakes@moffitt.org (J.M.F.); sarah.hoffe@moffitt.org (S.E.H.); jose.pimiento@moffitt.org (J.M.P.); 2 Department of Thoracic Oncology, H. Lee Moffitt Cancer Center, Tampa, FL 33612, USA; jobelle.baldonado@moffitt.org (J.J.B.); jacques.fontaine@moffitt.org (J.P.F.)

**Keywords:** induction chemotherapy, neoadjuvant therapy, gastroesophageal adenocarcinoma, response to therapy

## Abstract

**Simple Summary:**

Induction chemotherapy followed by PET-adapted consolidation chemoradiotherapy has been ex-plored with success in the clinical trial setting for patients with high-risk gastroesophageal adeno-carcinoma. This study demonstrates that this strategy can be associated with high rate of pathologic complete response (pCR), sterilization of lymph node basin (ypN0), and margin-negative resection (R0) in high risk patients. Overall survival is favorable for patients who show metabolic response to 5-FU based chemotherapy and continue this during chemoradiation

**Abstract:**

**Background:** Neoadjuvant chemoradiation with esophagectomy is standard management for locally advanced esophageal adenocarcinoma. Induction chemotherapy with a tailored approach to chemoradiation based on metabolic response to therapy on PET was explored as an alternative strategy in the CALGB 80803 trial. We sought to describe real-world institutional experience implementing this approach outside of a clinical trial. **Methods:** Patients who were treated with induction fluorouracil-leucovorin-oxaliplatin (FOLFOX) or fluorouracil-leucovorin-oxaliplatin-docetaxel (FLOT) with tailored chemoradiation based on PET response and subsequent esophagectomy were identified from a prospectively maintained database. Primary outcomes were pathologic complete response (pCR) and overall survival (OS) following completion of all therapy. **Results:** There were 35 patients who completed induction chemotherapy, chemoradiation, and esophagectomy. Thirty-three completed restaging PET following induction chemotherapy with metabolic response seen in 76% (n = 25/33). The pCR rate was 31% (n = 11/35) and the ypN0 rate was 71% (n = 25/35). Among the patients who demonstrated metabolic response to induction FOLFOX/FLOT and subsequently continued fluorouracil-based chemoradiation, the pCR rate was 39% (n = 9/23). The rate of pathologically negative lymph nodes in this group was high (n = 19/23, 83%) with 100% R0 resection rate (n = 23/23). With the median follow-up of 43 months, the median OS was not reached for this group and was significantly longer than the OS for the remainder of the cohort (*p* = 0.027, *p* = 0.046 adjusted for clinical stage). **Conclusions:** Induction FOLFOX/FLOT chemotherapy with evaluation of sensitivity via metabolic response and tailored chemoradiation seems to lead to high pCR and ypN0 rates in high-risk patients with adenocarcinoma of the esophagus and GE junction. This approach in clinical practice seems to recapitulate encouraging results in clinical trials.

## 1. Introduction

Neoadjuvant chemoradiation followed by surgery is the current standard management of esophageal cancer with evidence of locally advanced primary tumor and/or regional lymphadenopathy. This has been established in several prospective, randomized trials accruing patients with resectable <N2 disease, most notably, the CROSS trial, which demonstrated an improvement in overall survival for patients receiving neoadjuvant chemoradiation versus surgery alone [1,2]. Despite this, patients recur following chemoradiation and esophagectomy [3]. Moreover, few studies directly address outcomes in patients with higher risk disease such as >N1 nodal involvement. Adenocarcinoma in particular is known to be less radiosensitive than squamous cell carcinoma of the esophagus and may be at higher risk of recurrence [2,3] despite strategies to improve tumor response rates [3,4]. One of the major criticisms of chemoradiation is the lack of knowledge of sensitivity to the treatment until the patient has repeated imaging prior to planned surgery. Based on the early pioneering work on the MUNICON trial, the CALGB 80803 study was designed to assess if PET could be used as an early guide for response to induction therapy, and further chemoradiation can be modified if the desired response is not achieved [5]. Additionally, there are multiple chemoradiation regimens available for use with varying levels of efficacy on an individualized patient level.

In the CALGB 80803 study, patients were randomly assigned to receive induction chemotherapy with either modified oxaliplatin, leucovorin, and fluorouracil (FOLFOX) or carboplatin-paclitaxel (CP), and subsequently restaged with PET to assess the response. Patients then proceeded to chemoradiation using either FOLFOX or CP, depending on whether or not the tumor responded to this regimen during induction chemotherapy. Although not designed as a randomized phase III trial, it was observed that patients who received induction FOLFOX and had a metabolic response as defined by PET and continued on chemoradiation based on this regimen had excellent outcomes [6].

In light of these favorable results, our institution has adopted a practice of preferring induction fluorouracil-based therapy for esophageal adenocarcinoma that we consider high risk after a multidisciplinary tumor board review, with a tailored approach to chemoradiation depending on the PET response to the induction therapy. With the limited real-world data published on this novel approach to esophageal adenocarcinoma, we sought to describe our institutional experience with this multimodal treatment strategy. In this report, we focus on pathologic complete response and postoperative oncologic outcomes among patients treated with induction fluorouracil-based systemic chemotherapy for gastroesophageal adenocarcinoma with tailored chemoradiation, and subsequent esophagectomy. We also examine how these outcomes compare to established outcomes from prospective studies, including the CROSS and CALGB 80803 data.

## 2. Methods

**Figure 1:**
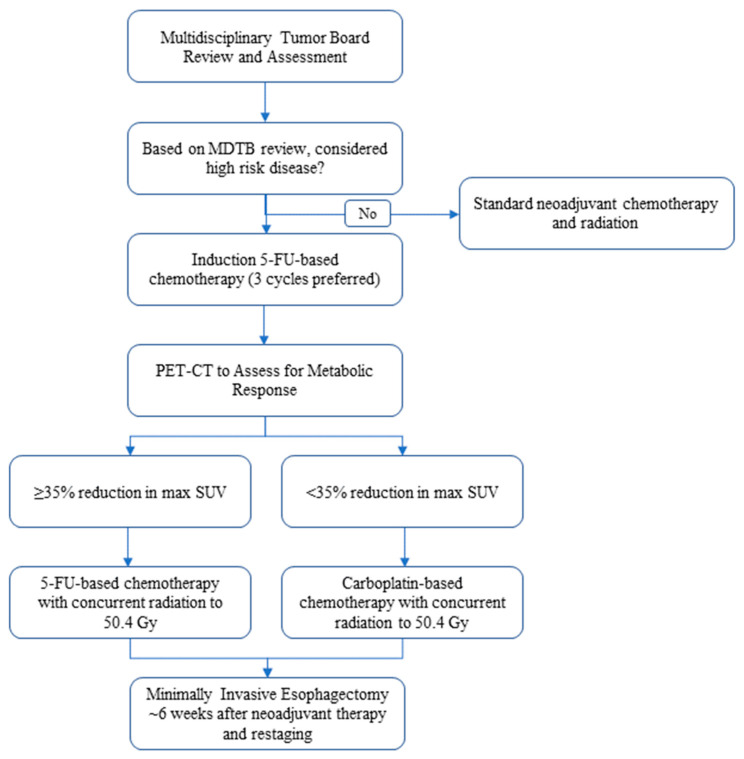
Protocol flow chart for use of induction chemotherapy.

This is a retrospective observational cohort study performed at a specialty cancer center using a prospectively maintained institutional database (2018–2022). Patients with nonmetastatic adenocarcinoma of the esophagus or gastroesophageal junction who were treated with induction fluorouracil-based chemotherapy, followed by concurrent chemoradiation and subsequent esophagectomy with curative intent, were identified. As informed by the CALGB 80803 protocol, institutional practice is to identify patients with adenocarcinoma felt to be at high risk of occult metastatic disease and/or recurrence after a multidisciplinary tumor board review. Following completion of induction chemotherapy, restaging PET is obtained. A response with a ≥35% decrease in maximum SUV is considered an adequate response to chemotherapy, and this regimen is continued as the backbone for chemoradiation. Radiation was routinely administered to a dose of 50.4 Gy given the preoperative intent. All surgical resections were performed at our primary institution, while neoadjuvant chemotherapy and radiation were either administered at our primary institution or in coordination with referring facilities. Esophagectomy was routinely performed using a transthoracic (Ivor Lewis) approach utilizing minimally invasive techniques (robotic assisted within the thoracic cavity). Figure 1 demonstrates the flow chart of the patients included in this cohort.

The primary outcomes of interest were the rate of pathologic complete response following a full course of multi-modality therapy as well as overall survival (OS). All surgical specimens were reviewed by a specialized gastrointestinal pathologist. Pathologic complete response was defined as no cancer cells seen in the primary luminal tumor or lymph nodes (ypT0N0). OS was defined as time from diagnosis to death from any cause with patients censored if alive at the time of last follow-up.

Covariates considered for analysis included clinical, pathologic, and treatment factors. Clinical variables were age, sex, and medical comorbidities. Tumor variables included primary tumor site (middle thoracic esophagus, distal thoracic esophagus, or gastroesophageal junction), clinical T stage, clinical N stage (classified as cN0, cN+, or cNx), and differentiation (well/moderate versus poor). Treatment variables considered included total radiation dose administered, cycles of induction chemotherapy administered. PET response following induction chemotherapy was considered complete if no residual FDG-avid disease was identified, good/partial if maximum SUV decreased by ≥35%, and poor if maximum SUV did not decrease by 35%.

Descriptive statistics are presented as frequency for categorical variables and median with interquartile range (IQR) for continuous variables. Univariate analyses were performed using Chi-square, Fisher exact, or Wilcoxon rank-sum tests, as appropriate. OS was analyzed using the Kaplan–Meier method and Cox proportional hazard regression. The log-rank test was used for univariate statistical testing for this time-to-event outcome. Multivariable Cox analysis was performed as possible within the constraints of event rate. Proportional hazard assumption was tested by assessing Schoenfeld residuals, and model goodness of fit was assessed using Cox–Snell residuals. The median follow-up time was assessed using the reverse Kaplan–Meier method [7]. All statistical analyses were performed using Stata version 14 (StataCorp. Stata Statistical Software: Release 14. College Station, TX, USA: StataCorp LP; 2015). This study was approved by the Institutional Review Board at Moffitt Cancer Center Protocol Number 15030. Patient consent was waived after IRB review.

## 3. Results

### 3.1. Study Cohort

There were 35 patients from the 2018–2022 period who were treated with induction fluorouracil-based chemotherapy, followed by tailored chemoradiation and subsequent esophagectomy (Table 1). All patients had biopsy-confirmed adenocarcinoma histology. Three (n = 3, 9%) patients with gastroesophageal junction tumors received induction FLOT, and the remainder (n = 32, 91%) received FOLFOX; both groups were included for the study. The median age was 63 years (IQR 59–69), and the majority were male (n = 32, 91%) and white (n = 33, 89%). The majority of patients were clinical stage III at diagnosis based on EUS and PET (n = 31, 88%). The clinical T stage was T3/T4 for 28 cases (80%), with clinically involved regional lymphadenopathy in 29 cases (83%).

### 3.2. Induction Chemotherapy and Chemoradiation Regimen

The number of induction chemotherapy cycles administered ranged from three to five, with the majority receiving either three (n = 23, 66%) or four cycles (n = 10, 29%). The median length of time from diagnosis to surgery was 6.9 months (IQR 6.0–8.5 months). Metabolic response to therapy was not assessed in two patients due to administrative issues with obtaining PET. Among the 33 patients who underwent restaging PET prior to chemoradiation, metabolic response with ≥35% reduction in maximum SUV was observed in 25 (76%) cases. Twenty-two of these patients remained on FOLFOX during chemoradiation, and one additional patient received fluorouracil-cisplatin due to significant toxicity to oxaliplatin during induction chemotherapy but with the intent to continue fluorouracil-based therapy. Eight patients (n = 8/35 23%) were changed to CP-based chemoradiotherapy. The rationale for the backbone change included documented poor response to induction therapy on PET (n = 5), severe toxicity from FLOT therapy (n = 1), and provider preference from referring physicians (n = 2). One patient had a complete clinical response evidenced by no residual identifiable tumor on EGD following induction FOLFOX and FOLFOX-based chemoradiation and elected to defer surgery; salvage esophagectomy was performed 30 months after initial diagnosis. The radiation dose was prescribed to 50.4 Gy in the majority of cases; however, there were a few patients who were uncertain if they would ultimately agree to resection so they received a simultaneous integrated boost to the gross tumor volume to 56 Gy, which was previously shown to be safe and effective in our practice [4].

Postoperative outcomes are shown in Table 2. All resections were performed with a minimally invasive approach, with all but one case performed with a robotic-assisted Ivor Lewis esophagectomy; the remaining case was performed as a robotic-assisted three-hole esophagectomy. The most common complication was postoperative atrial fibrillation, occurring in 10 patients (29%). The in-hospital leak rate was 9%, which is not higher than our previously published postoperative anastomotic leak rate. There were no postoperative mortalities within 30 days. The median length of postoperative stay was 8 days (IQR 7–9 days).

### 3.3. Pathologic Response following Induction Chemotherapy and Chemoradiation

Among the full cohort of patients who received induction FOLFOX/FLOT, consolidation chemoradiation, and subsequent esophagectomy, the rate of pCR overall was 31% (n = 11/35). With a median of 23 lymph nodes removed with esophagectomy (range 11–43), 71% of cases were found to have no disease in the regional nodes (ypN0; n = 25/35). Among patients who demonstrated a metabolic response to induction therapy and subsequently continued fluorouracil-based therapy during chemoradiation (FOLFOX n = 22, fluorouracil-cisplatin n = 1), the rate of pCR was 39% (n = 9/23). The rate of pathologically negative regional lymph nodes in this group was high (n = 19/23, 83%) with a 100% R0 resection rate (n = 23/23). In contrast, the pCR rate among PET non-responders was 25% (n = 2/8) and the ypN0 rate was 50% (n = 4/8). These differences did not reach statistical significance likely due to the small sample size (*p* = 0.45 and *p* = 0.068, respectively). Based on the year of diagnosis and the presence of residual disease after esophagectomy, eight patients were potential candidates for adjuvant immunotherapy. Six patients received adjuvant nivolumab therapy and two declined. Immunotherapy was discontinued early for two patients due to poor tolerance (n = 1) or disease progression (n = 1).

### 3.4. Overall Survival from First Diagnosis

**Figure 2:**
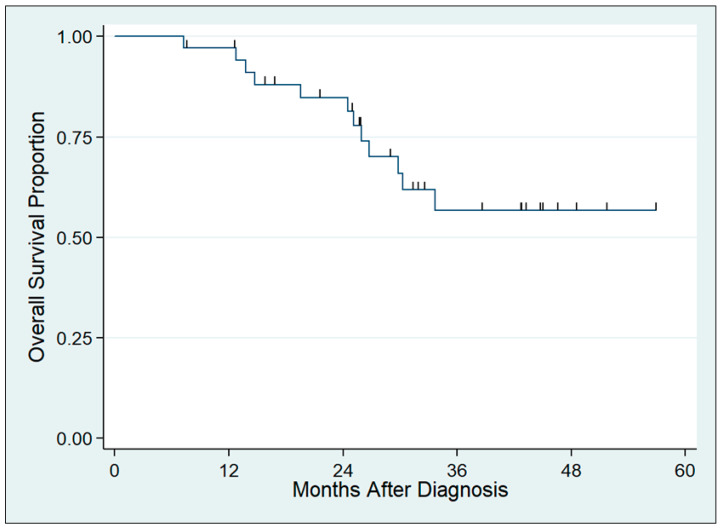
Overall survival for full cohort, from diagnosis to death or last follow-up.

**Figure 3:**
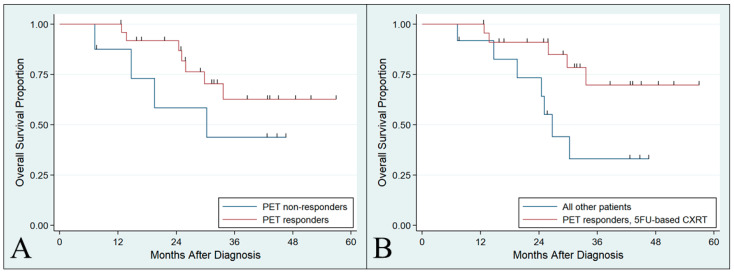
Overall survival among cohort subgroups. (**A**) Patients who responded to induction FOLFOX/FLOT were observed to have favorable survival versus non-responders, but this was not statistically significant (*p* = 0.23). (**B**) Responders to induction FOLFOX/FLOT who subsequently received fluorouracil-based chemoradiation had favorable OS compared to the remainder of the cohort (*p* = 0.027). These results remained unchanged excluding patients with salvage esophagectomy (*p* = 0.029).

The median follow-up time was 43 months, during which time there were 12 deaths (Figure 2). The 3-year OS for the full cohort was 57.0% (95% CI 36.5–73.1%). Metabolic response following induction chemotherapy was observed to have favorable survival compared to non-response, but this was not statistically significant (Figure 3A, median OS not reached versus 30 months, *p* = 0.23). Patients who demonstrated metabolic response to induction therapy and subsequently received fluorouracil-based chemoradiation (n = 23) had significantly favorable OS compared to all other patients in the cohort (Figure 3B, median OS not reached versus 27 months, *p* = 0.027). This remained statistically significant after adjustment for AJCC stage (HR 0.31, *p* = 0.046). Additional multivariable adjustment was not possible due to a few events.

## 4. Discussion

While neoadjuvant chemoradiation for locoregional esophageal and GE junction adenocarcinoma remains the standard of care, strategies to improve oncologic outcomes for patients have included the use of chemotherapy prior to chemoradiotherapy. CALGB 80803 showed an approach that aligns to early determination of sensitivity to induction chemotherapy based on the use of metabolic PET response with further tailoring of the chemoradiation backbone leading to favorable oncologic outcomes. We present here a cohort of 35 patients treated following these principles. Notably, our cohort comprises high-risk individuals, mostly with bulky T3 or T4 adenocarcinomas and/or with multiple positive lymph nodes. While these selection criteria do not strictly follow the original CALGB 80803 design, our main motivation was to be able to better define sensitivity to treatment early in the therapy process.

In this cohort, metabolic response to induction FOLFOX/FLOT therapy as measured by PET-CT was seen at a rate of 76%. Pathologic complete responses were seen in 31% of all patients, with a high rate of pathologically negative lymph nodes (ypN0) at 71% following induction chemotherapy and consolidation chemoradiation—notable given the 83% clinically node-positive rate in the cohort. In metabolic responders to induction FOLFOX/FLOT who continued fluorouracil-based chemoradiation, pathologic findings at surgery were promising, with a pCR rate of 39%, ypN0 rate of 83%, and R0 rate of 100%. Furthermore, these patients were observed to have favorable OS with median survival not yet reached. This was significantly higher than the other patients in the cohort. A longer-term follow-up will be valuable for determining if these outcomes remain.

The metabolic response rate to FOLFOX induction therapy in the CALGB 80803 was about 65%, which is less than reported in this study [6]. This can be attributed to the smaller sample size and patients being treated at the same center. There were about 90% of patients in CALGB 80803 that had moderately or poorly differentiated tumors, while our study had 71% of tumors classified as such. The finding of a pCR rate of 39% for patients who responded to induction therapy and continued 5FU-based chemoradiotherapy is consistent with CALGB 80803 results, which observed a pCR rate of 40% in this group. It is important to remember that patients in this cohort exclusively had adenocarcinoma, which is inherently less sensitive to chemoradiation than squamous cell carcinoma. In comparison, the rate of pCR in the CROSS trial using a CP-based approach was 23% for adenocarcinoma [1]. Furthermore, this cohort had a high rate of cN+ disease at 83%, compared to 65% in the CROSS trial. (2) Despite this, the rate of ypN0 after induction therapy and adapted chemoradiotherapy for responders was similar to the 69% seen in CROSS, at 71%. One might expect a lower rate of ypN0 disease if starting with a higher frequency of clinically suspicious regional lymph node disease. Additionally, the finding of the 3-year OS of 57% is notable given that the cohort consisted primarily of patients with clinically T3 or T4 tumors with a high burden of involved lymph nodes, very similar to the 3-year OS for adenocarcinoma patients treated with neoadjuvant therapy in CROSS based on visual approximation of the published survival curves. (2) In comparison, the 5-year OS for the subset of patients responding to induction FOLFOX and continuing FOLFOX-based chemoradiation in CALGB 80,803 was 53% [6]. We feel our that limited experience suggests that these encouraging results may be replicated outside of the context of a clinical trial.

Importantly, some hurdles that were encountered during the application of the protocol in clinical practice included the impossibility of obtaining the intended PET scan following induction chemotherapy due to insurance denial in 2 out of 35 cases (6%). This brings to light an important factor that must be considered—that adopting this approach may require significant effort by the clinician for obtaining insurance approval while this approach is not considered to be standard of care. This may limit its widespread use if the appropriate support is not available. The CALGB 80803 study is an important study that highlights the importance of using PET response to tailor chemoradiation after induction chemotherapy, and we continue to encourage the use of this strategy in our institutional pathways. Additionally, it was observed that some referring providers preferred the use of CP-based chemoradiation resulting in patients being transitioned to this type of therapy despite demonstrating a metabolic response to FOLFOX induction therapy. In the current time of chemotherapy shortages especially with carboplatin and cisplatin, the importance of this chemotherapy combination is now increasingly pertinent.

The addition of induction chemotherapy prolonged the neoadjuvant therapy to a median of 6.9 months, as compared to the typical 5-week course with a 4–6-week delay to surgery. This parallels the trend toward “total neoadjuvant therapy” seen in other disease sites including rectal and pancreas [8,9,10,11,12]. This could be considered a downside; however, this might also allow occult stage IV patients to declare themselves prior to undergoing a highly morbid surgery with the dreaded outcome of early postoperative distant recurrence. We cannot comment on this specifically as we do not have a denominator of patients who dropped out during therapy, and hopefully this will be clarified as experience with this approach to therapy grows. However, it should be acknowledged that this prolonged period of therapy may have increased toxicity from chemotherapy. Perceived advantages to induction therapy include early initiation of therapy which may prevent clinical deterioration, notably with potential worsening of dysphagia and the need for enteral access. It also becomes possible to ensure a sensitive chemotherapy backbone before pursuing chemoradiation.

There are notable limitations to this study. While OS was favorable among patients with metabolic response to induction therapy, there is an element of selection bias in this analysis. However, this is still valuable information as we strive for the best possible outcomes for individual patients. In addition to this selection bias, it should be acknowledged that the study was performed at a high-volume, experienced center. It is possible that these favorable survival outcomes may not be replicated in lower-volume centers. In particular, postoperative outcomes in this cohort were favorable, likely reflecting the experience with high volumes. Surgical resections were routinely performed using a minimally invasive approach, which is rapidly becoming the standard of care. It should additionally be noted that patients in this study were diagnosed from the 2018–2022 period. Adjuvant immunotherapy is now routinely offered to patients with residual disease following chemoradiation (i.e., those without pCR) as this has been shown to prolong recurrence-free survival [13]. Given the period of study, this was not available to most patients in this cohort and may have impacted longer-term oncologic outcomes.

While this study provides some promising results for the potential use of induction chemotherapy prior to chemoradiation, some particular points should be noted. Future studies will be valuable for better defining the role of induction chemotherapy in the context of evolving standard of care and other available treatment modalities. For example, it is increasingly appreciated that MSI-high and MMR-deficient tumors appear to be responsive to immunotherapies, with a less favorable response to conventional cytologic chemotherapy. Importantly, it should be noted that no cases in this cohort had an MSI-high or MMR-deficient biology, which might alternatively be responsive to an immunotherapy-based approach. The results of the current study, therefore, should not be applied to patients with these types of tumors. Additionally, while neoadjuvant chemoradiation remains the preferred approach for the management of Siewert I and II tumors in the United States, FLOT perioperative chemotherapy is an acceptable approach to the management of gastroesophageal junction adenocarcinoma and may be preferred in Europe. The results of the current study are too limited to support the addition of radiotherapy to this approach. Current ongoing trials should enlighten us soon regarding the optimal approach to GE junction adenocarcinomas.

## 5. Conclusions

Induction chemotherapy with evaluation of sensitivity by metabolic response and tailored chemoradiation aligning to recent prospective trial regimens may translate to the real-world setting and lead to high rates of complete pathologic response and sterilization of locoregional nodal basins in high-risk patients with adenocarcinoma of the esophagus and GE junction. This approach validates the encouraging results in clinical trials and may provide a personalized treatment strategy for high-risk patients.

## Figures and Tables

**Table 1:** Clinical, tumor, and treatment characteristics of the cohort, n = 35.

Patient Factors	
Age, years (median (IQR))	63 (59–69)
Male	32 (91)
Race	
White	33 (89)
Hispanic	3 (9)
African American	1 (3)
Karnofsky Performance Status, median (IQR)	90 (80–90)
Charlson Comorbidity Index, median (IQR)	1 (0–1)
**Tumor Factors**	
Stage *	
IIA	2 (6)
IIB	2 (6)
III	31 (88)
Primary Tumor Site	
Middle esophagus	1 (3)
Distal esophagus	20 (57)
Gastroesophageal junction	14 (40)
Adenocarcinoma histology	35 (100)
Tumor Differentiation	
Well	2 (5)
Moderate	12 (32)
Poor	15 (39)
Unspecified	9 (24)
Her2-positive ^a^	9 (50)
MSI high ^b^	0 (0)
**Treatment Factors**	
Induction regimen	
FOLFOX	32 (91)
FLOT	3 (9)
Cycles of induction chemotherapy	
3	23 (66)
4	10 (29)
5	2 (6)
Radiotherapy dose	
50.4 Gy	30 (86)
56 Gy	5 (14)
Chemoradiation Backbone	
FOLFOX	26 (74)
Carboplatin-Paclitaxel	8 (23)
Fluorouracil-cisplatin **	1 (3)
Operative approach	
Minimally invasive Ivor Lewis esophagectomy	34 (97)
Minimally invasive three-hole esophagectomy	1 (3)

* Five patients were unable to complete full endoscopic ultrasound staging due to an obstructing tumor but were considered at least locally advanced based on imaging. ** One patient with sensitivity to oxaliplatin during induction chemotherapy was treated with fluorouracil-cisplatin. ^a^ Data available for 18 cases. ^b^ Data available for 11 cases.

**Table 2:** Postoperative surgical outcomes following induction chemotherapy and consolidation chemoradiation.

Outcome	n (%)
Pneumonia	2 (6)
Myocardial infarction	0 (0)
Postoperative atrial fibrillation	10 (29)
In-house anastomotic leak	3 (9)
Transfer to Intensive Care Unit	2 (6)
Mortality within 30 days	0 (0)
Length of stay, days (median (IQR))	8 (7–9)

## Data Availability

The data set for this study is available from the authors upon reasonable request (AJS or JMP).

## References

[1] van Hagen P., Hulshof M.C., van Lanschot J.J., Steyerberg E.W., van Berge Henegouwen M.I., Wijnhoven B.P., Richel D.J., Nieuwenhuijzen G.A., Hospers G.A., Bonenkamp J.J. (2012). Preoperative chemoradiotherapy for esophageal or junctional cancer. N. Engl. J. Med..

[2] Shapiro J., van Lanschot J.J.B., Hulshof M., van Hagen P., van Berge Henegouwen M.I., Wijnhoven B.P.L., van Laarhoven H.W.M., Nieuwenhuijzen G.A.P., Hospers G.A.P., Bonenkamp J.J. (2015). Neoadjuvant chemoradiotherapy plus surgery versus surgery alone for oesophageal or junctional cancer (CROSS): Long-term results of a randomised controlled trial. Lancet Oncol..

[3] Blackham A.U., SM H.N., Schell M.J., Jin W., Gangi A., Almhanna K., Fontaine J.P., Hoffe S.E., Frakes J., Venkat P. (2018). Recurrence patterns and associated factors of locoregional failure following neoadjuvant chemoradiation and surgery for esophageal cancer. J. Surg. Oncol..

[4] Venkat P.S., Shridhar R., Naghavi A.O., Hoffe S.E., Almhanna K., Pimiento J.M., Fontaine J.P., Abuodeh Y., Meredith K.L., Frakes J.M. (2017). Dose escalated neoadjuvant chemoradiotherapy with dose-painting intensity-modulated radiation therapy and improved pathologic complete response in locally advanced esophageal cancer. Dis. Esophagus.

[5] Lordick F., Ott K., Krause B.J., Weber W.A., Becker K., Stein H.J., Lorenzen S., Schuster T., Wieder H., Herrmann K. (2007). PET to assess early metabolic response and to guide treatment of adenocarcinoma of the oesophagogastric junction: The MUNICON phase II trial. Lancet Oncol..

[6] Goodman K.A., Ou F.S., Hall N.C., Bekaii-Saab T., Fruth B., Twohy E., Meyers M.O., Boffa D.J., Mitchell K., Frankel W.L. (2021). Randomized Phase II Study of PET Response-Adapted Combined Modality Therapy for Esophageal Cancer: Mature Results of the CALGB 80803 (Alliance) Trial. J. Clin. Oncol..

[7] Schemper M., Smith T.L. (1996). A note on quantifying follow-up in studies of failure time. Control. Clin. Trials.

[8] Truty M.J., Kendrick M.L., Nagorney D.M., Smoot R.L., Cleary S.P., Graham R.P., Goenka A.H., Hallemeier C.L., Haddock M.G., Harmsen W.S. (2021). Factors Predicting Response, Perioperative Outcomes, and Survival Following Total Neoadjuvant Therapy for Borderline/Locally Advanced Pancreatic Cancer. Ann. Surg..

[9] Donnelly M., Ryan O.K., Ryan É.J., Creavin B., O’Reilly M., McDermott R., Kennelly R., Hanly A., Martin S.T., Winter D.C. (2023). Total neoadjuvant therapy versus standard neoadjuvant treatment strategies for the management of locally advanced rectal cancer: Network meta-analysis of randomized clinical trials. Br. J. Surg..

[10] Bahadoer R.R., Dijkstra E.A., van Etten B., Marijnen C.A.M., Putter H., Kranenbarg E.M., Roodvoets A.G.H., Nagtegaal I.D., Beets-Tan R.G.H., Blomqvist L.K. (2021). Short-course radiotherapy followed by chemotherapy before total mesorectal excision (TME) versus preoperative chemoradiotherapy, TME, and optional adjuvant chemotherapy in locally advanced rectal cancer (RAPIDO): A randomised, open-label, phase 3 trial. Lancet Oncol..

[11] Conroy T., Bosset J.F., Etienne P.L., Rio E., François É., Mesgouez-Nebout N., Vendrely V., Artignan X., Bouché O., Gargot D. (2021). Neoadjuvant chemotherapy with FOLFIRINOX and preoperative chemoradiotherapy for patients with locally advanced rectal cancer (UNICANCER-PRODIGE 23): A multicentre, randomised, open-label, phase 3 trial. Lancet Oncol..

[12] Murphy J.E., Wo J.Y., Ryan D.P., Jiang W., Yeap B.Y., Drapek L.C., Blaszkowsky L.S., Kwak E.L., Allen J.N., Clark J.W. (2018). Total Neoadjuvant Therapy With FOLFIRINOX Followed by Individualized Chemoradiotherapy for Borderline Resectable Pancreatic Adenocarcinoma: A Phase 2 Clinical Trial. JAMA Oncol..

[13] Kelly R.J., Ajani J.A., Kuzdzal J., Zander T., Van Cutsem E., Piessen G., Mendez G., Feliciano J., Motoyama S., Lièvre A. (2021). Adjuvant Nivolumab in Resected Esophageal or Gastroesophageal Junction Cancer. N. Engl. J. Med..

